# A super-grayscale and real-time computer-generated Moiré profilometry using video grating projection

**DOI:** 10.1038/s41598-021-99420-8

**Published:** 2021-10-06

**Authors:** Hongmei Li, Yiping Cao, Yingying Wan, Chengmeng Li, Cai Xu, Hechen Zhang, Haihua An

**Affiliations:** 1 Department of Optical Electronics, Sichuan University, Chengdu, 610064 China; 2 College of Physics and Engineering, Chengdu Normal University, Chengdu, 611130 China

**Keywords:** Applied optics, Optical techniques

## Abstract

By using the time-division multiplexing characteristics of the projector and the integral exposure characteristics of the charge coupled device (CCD) camera, a super-grayscale and real-time computer-generated Moiré profilometry based on video grating projection is proposed. The traditional digital static grating is of 256-grayscale at most. If an expected super-grayscale grating with a maximum grayscale of 766 is designed and divided into three 256-grayscale fringe patterns with balanced grayscale as far as possible, they can be synthesized into a repeated playing video grating instead of the traditional static grating. When the video grating is projected onto the measured object, as long as the exposure time is set to three times the refresh cycle of the video grating, the super-grayscale deformed patterns in the 766-grayscale can be captured with a 10-bit CCD camera, so that the deformed patterns are realistic. The digital error in computer-generated Moiré profilometry is effectively reduced. In addition, this method can expand the linear range of the deformed pattern by 20% in computer Moiré profilometry. Therefore, the proposed method has the perspectives of high accuracy and real-time measurement. Theoretical analysis and experimental results demonstrate the validity and capability of the proposed method.

## Introduction

Optical three-dimensional (3D) technology^1–5^ has been widely used in various industries such as automobile production, quality inspection, intelligent traffic management, virtual reality, etc., for the merits of non-contact, high speed, high spatial resolution, full-field measurement, and high precision^6–8^. Active optical 3D measurement technology^9–11^ adopts the method of active coding of structured light and uses digital projector to project it onto the surface of the measured object. A charge coupled device (CCD) can capture the deformed pattern which is modulated by the measured object and reconstruct the 3D surface after computer processing^12,13^. Due to its advantages of easy implementation, fast measurement speed and high precision, various methods have been developed for active optical 3D technology.

Phase Measuring Profilometry (PMP)^14,15^ is one of the most representative active optical 3D measurement methods. It can calculate the phase map of the measured object from multiple (at least three) phase-shifting fringe patterns^16,17^. The measurement accuracy of the PMP may be of the highest in the active optical 3D measurement, and it has a strong robustness to the ambient light for measuring static objects. However, for its multiple-shots feature, the measurement accuracy would be descended when measuring the moving object. Consequently, it may not be the best suitable method for real-time measurement. Fourier Transform profilometry (FTP)^18–20^, as another specific category, the reconstructed surface is obtained by the procedure of fast Fourier transform, frequency domain filtering, inverse Fourier transform, phase extraction, unwrapped phase calculation, and phase-height mapping^21,22^. It is insensitive to motion disturbance due to its single-shot feature and is suitable for real-time measurement, but the measurement accuracy may be limited to some extent by the filtering operation.

Recently, C.M. Li et al. presented a computer-generated Moiré profilometry (CGMP)^23^. It has been proved to have the same single-shot characteristics as FTP, can be applied in real-time measurement, and have high measurement accuracy close to PMP. C.M. Li et al. proposed a high accuracy CGMP with π phase-shifting grating projection^24^. Lu. Wang et al. proposed two improved CGMP with orthogonal modulation techniques^25,26^. H.C. Zhang et al. proposed another improved CGMP with color composite grating projection^27^. All these methods can improve the accuracy of CGMP indeed. They are all based on structure light projection with a digital projector, and the projected grating is original of 256-grayscale at most due to the 256-grayscale standard of the digital projector. So the captured deformed pattern is of 256 grayscale at most.

In this study, by skillfully using the time-division multiplexing characteristics of the digital projector and the integration characteristics of the CCD camera, a super-grayscale, and real-time computer-generated Moiré profilometry (SRCGMP) is proposed. Firstly, an expected super-grayscale grating with 766-grayscale is designed on a computer; then, it is divided into three traditional 255-grayscale fringe patterns with balanced grayscale as far as possible. These three fringe patterns are synthesized into a repeated video, which is called a video grating. When the video grating, which replaces the traditional static grating, is projected onto the measured object, a 10-bit CCD camera can capture the deformed pattern with 766-grayscale if the exposure time of the CCD camera is set to three times the refresh period of the video grating. Compared with the traditional technology, the video grating projection has the advantages of getting the super-grayscale deformed pattern with less digital error and higher resolution phase information. Meanwhile, the linear range of the super-grayscale deformed pattern can be expanded by 20%. The measurement accuracy of CGMP is improved originally, and the single-shot feature of real-time measurement is maintained. The experimental results demonstrate the validity and capability of the proposed method.

## Principle

### Principle of super-grayscale fringe pattern capturing

Firstly, four expected super-grayscale gratings with 766-grayscale are designed as follows:

1 $$I_{n}^{766} (x,y) = a_{0}^{766} + b_{0}^{766} \cos [2\pi f_{0} x + 2(n - 1)\pi /4)]\quad n = 1,2,3,4$$

where $a^{766}_0$, $b^{766}_0$ are both normally set to be 382.5. The greyscale of each grating is in the scope of [0, 765]. $f_{0}$ is the spatial frequency of the four gratings. *n* is the number of the phase-shifting step number.

Due to the 256-grayscale standard of the commercial projector, the above super-grayscale gratings can’t be directly projected onto the measured object. Otherwise, it will automatically be compressed to 256-grayscale.

Based on the principle of time-division multiplexing, each grating is then divided into three 8-bit fringe patterns with 256-grayscale with balanced grayscale as far as possible:

2 $$\left\{ \begin{gathered} I_{n}^{766} (x,y) = I_{n1}^{256} (x,y) + I_{n2}^{256} (x,y) + I_{n3}^{256} (x,y) \hfill \\ \left| {I_{ni}^{256} (x,y) - I_{nj}^{256} (x,y)} \right| \le 1\quad i = 1,2,3;\;j = 1,2,3 \hfill \\ \end{gathered} \right.$$

**Figure 1:**
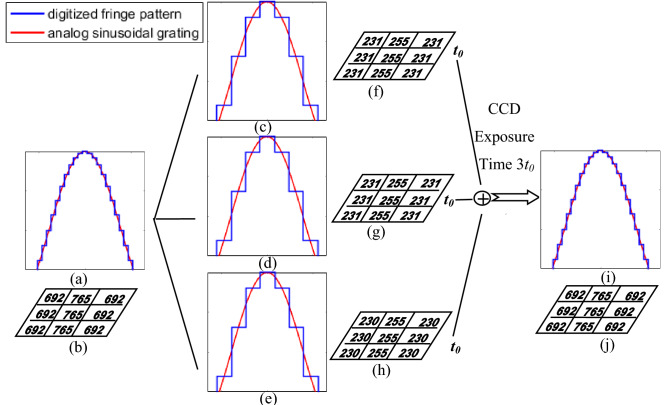
The division and time integration procession of the fringe pattern: (**a**) the cutaway view of the expected sinusoidal super-grayscale grating and analog sinusoidal grating; (**b**) gray values at partial pixels of the digitized grating in (**a**); (**c**–**e**)The cutaway views of the three divided fringe patterns with 256-grayscale and analog sinusoidal grating; (**f**–**h**) the corresponding gray values at partial pixels of digitized fringe patterns in (**c**–**e**); (**i**) the cutaway view of the captured super-grayscale pattern and analog sinusoidal grating; (**j**) the gray values at partial pixels of the digitized fringe pattern in (**i**).

The divided fringe patterns $I_{n1}^{256} (x,y),I_{n2}^{256} (x,y),I_{n3}^{256} (x,y)$ are then composed into a repeated playing video $vg_{n}$ which we called the video grating, and its refreshes cycle *t*_*0*_ is set to be consistent with the refresh cycle of the projector. Figure 1 gives the division and time integration procession of the fringe pattern. The more fitted between the digitized fringe pattern with the blue line and the analog sinusoidal grating with the red line, the more realistic the pattern information can be reflected.

The cutaway view of the expected super-grayscale sinusoidal grating and analog sinusoidal grating is shown in Fig. 1a. It shows that the digitized sinusoidal grating designed by the super-grayscale method is realistic because it is close to the expected analog sinusoidal grating. Figure 1b is gray values at partial pixels of the digitized grating in Fig. 1a. The cutaway views of the three corresponding divided fringe patterns with 256-grayscale $I_{n1}^{256} (x,y),I_{n2}^{256} (x,y),I_{n3}^{256} (x,y)$ and the analog sinusoidal grating are shown in Fig. 1c–e. The cutaway views show that they all deviate from the expected analog sinusoidal grating compared to that of the expected super-grayscale grating. Figure 1f–h are the corresponding gray values at partial pixels of digitized 256-grayscale fringe patterns in Fig. 1c–e. After setting the exposure time of the camera to be three times the refresh cycle (3 *t*_0_) of $vg_{n }$, and controlling the gain $A_{m}^{{}}$ of the camera appropriately, a 10-bit CCD camera can capture the deformed super-grayscale patterns with 766-grayscale by making full use of the integral characteristics of the camera, which can be expressed as follows:

3 $$\begin{aligned} I_{n}^{c766} (x,y) & = A_{m} [I_{n1}^{256} (x,y)t_{0} + I_{n2}^{256} (x,y)t_{0} + I_{n3}^{256} (x,y)t_{0} ] \\ & = A_{m} t_{0} [I_{n1}^{256} (x,y) + I_{n2}^{256} (x,y) + I_{n3}^{256} (x,y)] \\ \end{aligned}$$

The cutaway view of the captured super-grayscale pattern and the analog sinusoidal grating are shown in Fig. 1i, and Fig. 1j shows the gray values at partial pixels of the digitized fringe pattern. It shows that the expected super-grayscale pattern can be achieved, which is realistic and is helpful to improve the measurement accuracy originally.

### Principle of SRCGMP

By projecting each of the four video gratings onto the measuring object instead of the traditional static grating, and setting the exposure time of the CCD camera to three times the refresh period of the video grating, and controlling the gain of the camera properly, the four corresponding super-grayscale deformed patterns $I_{n}^{C766}$ can be captured with a 10-bit CCD camera. Since the calculations below are based on the SRCGMP measurement process, the formula below omits the superscript 766 for simplicity. The captured super-grayscale patterns can be expressed as:

4 $$I_{n}^{C} (x,y) = R_{0} (x,y)\left\{ {a_{0}^{{}} + b_{0}^{{}} \cos [2\pi fx + 2(n - 1)\pi /4 + \phi_{0} (x,y)} \right\}\quad n = 1,2,3,4$$

where $R_{{0}} (x,y)$ stands for the reflectivity of the reference plane. $\phi_{0} (x,y)$ denotes the phase distribution modulated by the reference plane. *f* is the spatial frequency of the fringe pattern captured from the reference plane.

One A.C. component of $I_{1}^{C} (x,y)$ can be obtained by:

5 $$I_{1}^{r} (x,y) = \frac{1}{2}(I_{1}^{C} - I_{3}^{C} ) = \frac{1}{2}R_{0} (x,y)b_{0} \cos [2\pi fx + \phi {}_{0}(x,y)]$$

The other AC component of $I_{{2}}^{C} (x,y)$ can be obtained by:

6 $$I_{2}^{r} (x,y) = \frac{1}{2}(I_{2}^{C} - I_{4}^{C} ) = \frac{1}{2}R_{0} (x,y)b_{0} \sin [2\pi fx + \phi {}_{0}(x,y)]$$

These two AC components of the reference plane with a π/2 phase difference have been prepared and stored in the computer in advance.

In measuring, only one video grating $vg_{1}$ is projected onto the measured object. And only one corresponding super-grayscale deformed pattern is needed to be captured as:

7 $$I_{o}^{C} (x,y) = R(x,y)\{ a_{0} + b_{0} \cos [2\pi fx + \phi (x,y)]\}$$

where $R(x,y)$ stands for the reflectively of the measured object, $\phi (x,y)$ denotes the phase distribution modulated by both the measurement object and the reference plane.

The DC component $R(x,y)a_{0}$ of captured deformed pattern distorted by the measured object can be filtered out in the frequency domain, and the AC component left can be written as follows:

8 $$\begin{aligned} I_{o}^{r} (x,y) & = R(x,y)b_{0} \cos [2\pi fx + \phi (x,y)] \\ & = I_{o}^{C} (x,y) - abs\left\{ {FFT^{ - 1} \left[ {FFT\left( {I_{o}^{C} (x,y)rect(x/f_{x} ,y/f_{y} )} \right)} \right]} \right\} \\ \end{aligned}$$

where $f_{x}$ denotes the width of the filter window along *x* direction and $f_{y}$ denotes the width of the filter window along the *y* direction. When this AC component multiplied by the two previously prepared AC components respectively, the results can be written as:

9 $$\begin{aligned} I_{0}^{or} (x,y) & = I_{o}^{r} (x,y)I_{1}^{r} = \frac{1}{2}R_{0} (x,y)R(x,y)b_{{_{0} }}^{2} \cos [4\pi fx + \phi_{0} (x,y) + \phi (x,y)] \\ & \quad + \frac{1}{2}R_{0} (x,y)R(x,y)b_{0}^{2} \cos [\phi (x,y) - \phi_{0} (x,y)] \\ \end{aligned}$$

10 $$\begin{aligned} I_{90}^{or} (x,y) & = I_{o}^{r} (x,y)I_{2}^{r} = \frac{1}{2}R_{0} (x,y)R(x,y)b_{{_{0} }}^{2} \sin [4\pi fx + \phi_{0} (x,y) + \phi (x,y)] \\ & \quad + \frac{1}{2}R_{0} (x,y)R(x,y)b_{0}^{2} \sin [\phi (x,y) - \phi_{0} (x,y)] \\ \end{aligned}$$

The second terms in Eq. (9) and Eq. (10) can be obtained by the low-pass filter, which are so-called computer-generated Moiré fringes. They can be written as:

11 $$I_{0}^{Moire} (x,y) = \frac{1}{2}R_{0} (x,y)R(x,y)b_{0}^{2} \cos [\phi (x,y) - \phi_{0} (x,y)]$$

12 $$I_{90}^{Moire} (x,y) = \frac{1}{2}R_{0} (x,y)R(x,y)b_{0}^{2} \sin [\phi (x,y) - \phi_{0} (x,y)]$$

Both Eqs. (11) and (12) contain the phase distribution only modulated by the measured object $\phi(x,y)-\phi_0 (x,y)$, which can be obtained as:

13 $$\phi (x,y) - \phi _{0} (x,y) = \text{arc} \;tan\left[ {I_{{90}}^{{Moire}} (x,y)/I_{0}^{{Moire}} (x,y)} \right]$$

Due to the arctangent operation, the phase distribution of the measured object is wrapped within $( - \pi ,\pi ]$. The unwrapped phase $\phi (x,y) - \phi_{0} (x,y)$ can be obtained by the phase unwrapping method^28^ to be $\Delta \Phi (x,y)$. The height information can be calculated by the height-phase mapping algorithm^29^:

14 $$\frac{1}{h(x,y)} = \alpha (x,y) + \beta_{1} (x,y)\frac{1}{\Delta \Phi (x,y)} + \beta_{2} (x,y)\frac{{\phi_{0} (x,y)}}{\Delta \Phi (x,y)} + \gamma (x,y)\frac{1}{{\Delta \Phi^{2} (x,y)}}$$

where the four coefficients of $a(x,y)$, $\beta_{1} (x,y)$, $\beta_{2} (x,y)$ and $\gamma (x,y)$ are system parameters, and they can be obtained by calibration.

The measurement accuracy can be improved originally for the phase resolution of the super-grayscale deformed pattern is more realistic than that of the traditional deformed pattern. Moreover, the single-shot feature is also retained. It has a prospective application for real-time measurement.

### Analysis of the linear relationship between the captured grayscale and the projected grayscale

**Figure 2:**
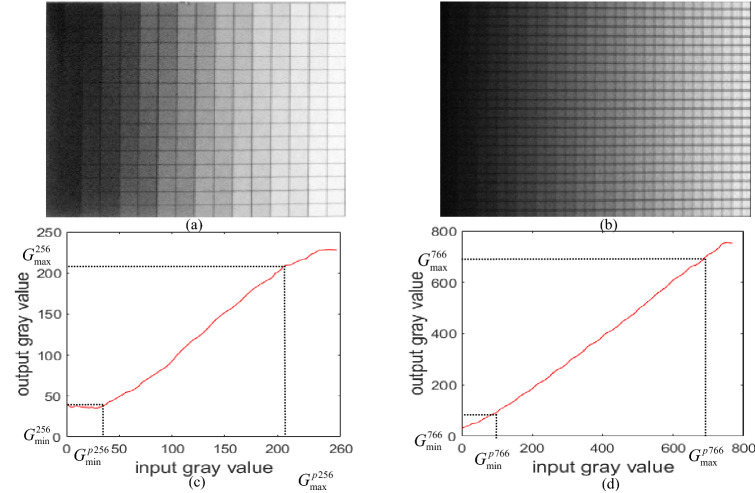
Linearly proportional analysis between traditional grayscale method and super-grayscale method: (**a**) captured pattern with traditional grayscale method; (**b**) captured pattern with super-grayscale method; (**c**) linear relationship between input gray value and output gray value in traditional grayscale method; (**d**) linear relationship between output gray value and input gray value in the super-grayscale method.

In order to investigate the linear relationship between captured grayscale and projected grayscale by the traditional method and the super-grayscale method, respectively, the following experimental steps are designed:

1. A pattern consisting of 16 × 16 grayed blocks is designed. Each grayed block contains a different gray level within [0, 255]. After it is projected onto the reference plane, the corresponding pattern is captured as Fig. 2a;
2. An expected 766-super-grayscale pattern consisting of 24 × 32 grayed blocks is designed. Each grayed block contains different gray level within [0, 765];
3. This expected 766-super-grayscale pattern is divided into three patterns with 256-grayscale according to Eq. (2), and then the three divided patterns are composed into a repeated playing video;
4. The repeated playing video is projected onto the reference plane, and a 766-super-grayscale pattern can be captured as shown in Fig. 2b as long as setting the exposure time of the camera to three times the refresh cycle of the video.
5. By calculating the gray values of each grayed block in the two captured patterns separately, the relationships between the output gray value and the input gray value in the traditional and super-grayscale methods can be figured out as shown in Fig. 2c,d, respectively.

Due to the nonlinear effect of the projector^30^, there are nonlinear phenomena in both methods. The linear interval of the grayscale using the traditional grayscale method is 38 to 210, the proportion of the linear interval is only about 67% with a linear regression coefficient of 0.9984. While the linear interval of the grayscale using the super-grayscale method is 128 to 738, the proportion of the linear interval can be expanded up to 80% with a better linear regression coefficient of 0.9997. It can be seen that the super-grayscale method has a stronger linear relationship between the input gray value and output gray value than that of the traditional method, and the linear range of captured pattern with the super-grayscale method can expand by 20%.

### Principle of optimized SRCGMP

In order to reduce the nonlinear influence of the digital projector on the captured deformed pattern, the four designed super-grayscale gratings, as shown in Eq. (1), are optimized. That is to ensure the peak grayscale and valley grayscale of sinusoidal gratings are in the linear range of super-grayscale grating.

The maximal and the minimal gray levels of the linear interval of the captured super-grayscale pattern are $G_{\max }^{766}$ and $G_{\min }^{766}$ respectively as shown in Fig. 2d, so:

15 $$I_{n}^{{766}} \left( {x,y} \right)_{{\min }} = a_{0}^{{766}} - b_{0}^{{766}} \ge G_{{\min }}^{{766}}$$

16 $$I_{n}^{{766}} \left( {x,y} \right)_{{\max }} = a_{0}^{{766}} + b_{0}^{{766}} \le G_{{\max }}^{{766}}$$

If the above conditions are satisfied and the linear section is fully utilized, the $a_{{{\text{0}}}}^{766}$ and $b_{{{\text{0}}}}^{766}$ can be calculated as:

17 $$a_{0}^{766} = \frac{{G_{\max }^{766} + G_{\min }^{766} }}{2}$$

18 $$b_{0}^{766} = \frac{{G_{\max }^{766} - G_{\min }^{766} }}{2}$$

In this way, the designed $a_{{{\text{0}}}}^{766}$ and $b_{{{\text{0}}}}^{766}$ can guarantee the captured super-grayscale pattern within the linear interval, and the wide grayscale range of the super-grayscale patterns can also be achieved. So the four expected super-grayscale gratings can be optimized as:

19 $$I_{n}^{O} (x,y) = \frac{{G_{\max }^{766} + G_{\min }^{766} }}{2} + \frac{{G_{\max }^{766} - G_{\min }^{766} }}{2}\cos [2\pi f_{0} x + 2(n - 1)\pi /4)]\quad n = 1,2,3,4$$

Each optimized grating is divided into three fringe patterns according to Eq. (2) for compositing the corresponding optimized repeated video. The four optimized video gratings are used instead of the previously designed four video gratings for projection. The optimized super-grayscale deformed patterns can be captured. After extracting and calculating the computer-generated Moiré fringes, the wrapped phase of the measured object can be obtained, and the height of the measured object can be efficiently reconstructed by phase-unwrapping and height-phase mapping. In this way, the measurement accuracy of the proposed SRCGMP can be improved further.

## Experimental results and analysis

**Figure 3:**
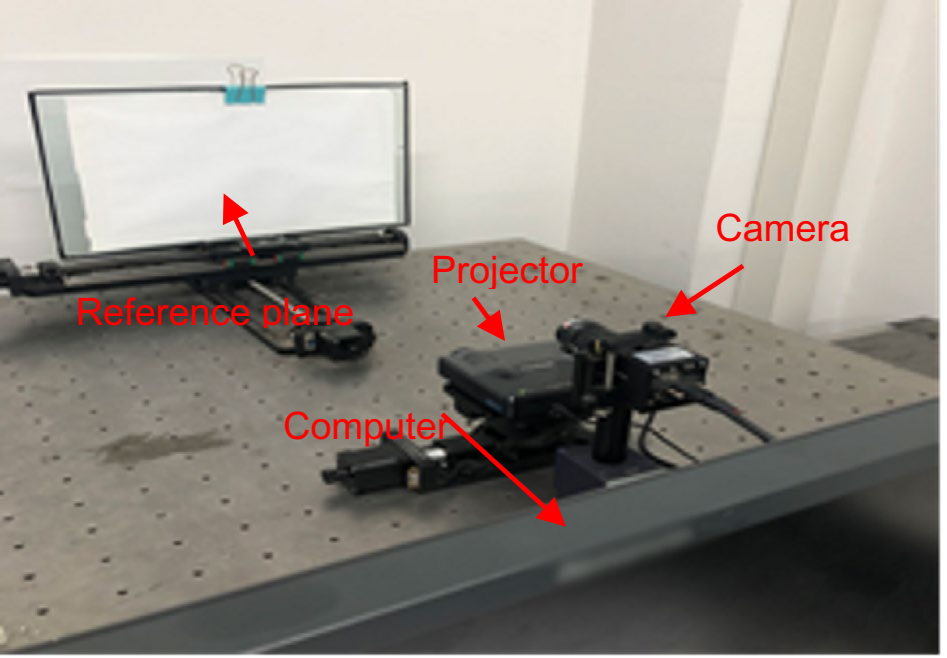
Experimental setup.

To verify the feasibility and validity of the proposed method, several experiments have been implemented. The experimental setup is shown in Fig. 3. It contains one projector (View Sonic PLED-W200) with a spatial resolution of 1200 × 800, and the maximum refresh rate is 75 fps. One camera (BEV-B1610 M) with a resolution of 1628 × 1236, which includes 8-bit, 10-bit, 12-bit, 14-bit four transmissions, and the 10-bit mode is used in the experiments; and one computer (Intel(R) Core (TM) i5-4590) is used for processing the information.

### The measurement accuracy between CGMP and SRCGMP

**Figure 4:**
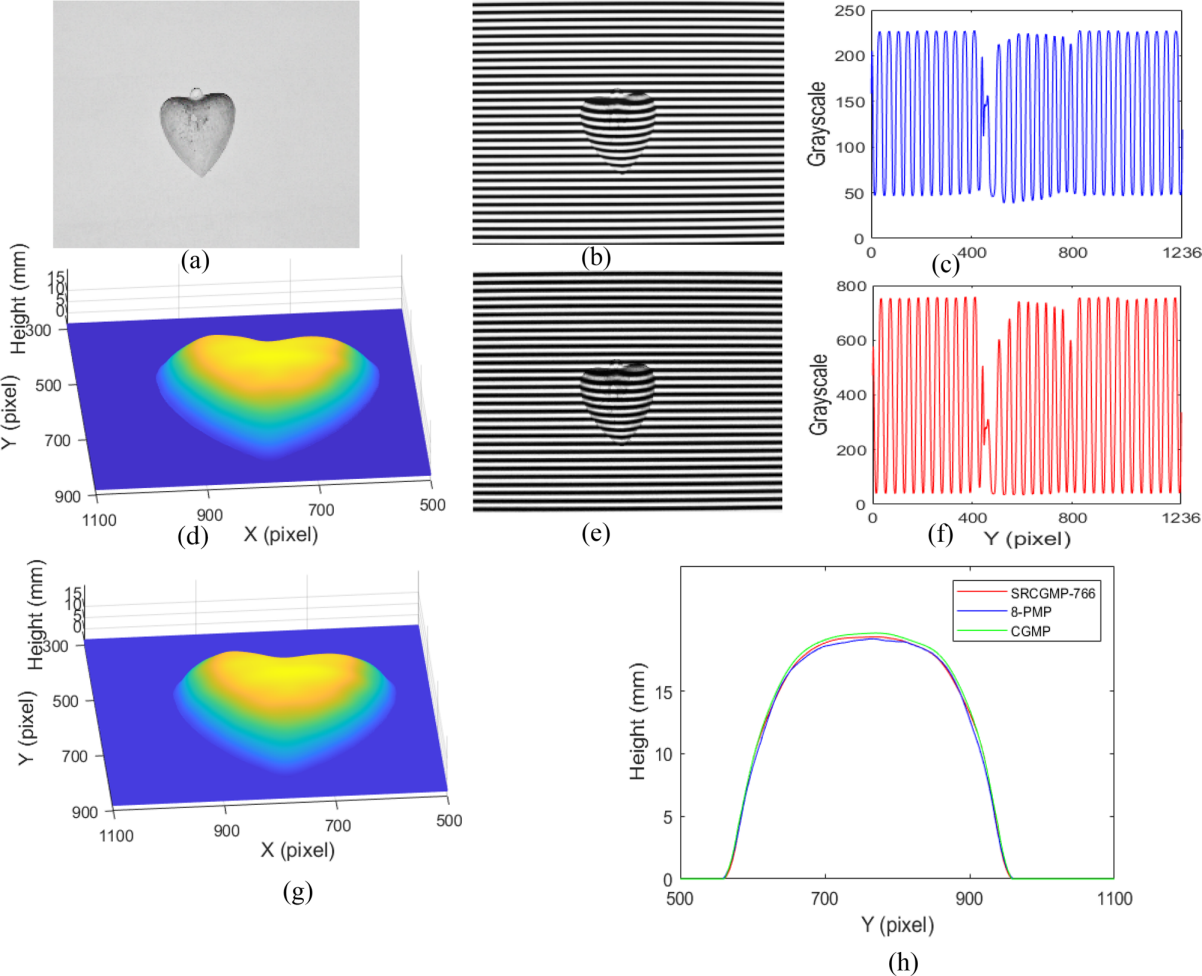
Measuring results of heart model: (**a**) the measured heart model; (**b**) the deformed pattern with CGMP; (**c**) the grayscale at the middle row of (**b**); (**d**) the reconstructed heart surface with CGMP; (**e**) the deformed pattern with SRCGMP-766; (**f**) the grayscale at the middle row of (**e**); (**g**) the reconstructed heart surface with SRCGMP-766; (**h**) the cutaway view at row 589 with the methods of SRCGMP-766, 8-PMP, and CGMP.

One heart model is measured to verify the validity of the SRCGMP with 766-grayscale (SRCGMP-766) and the measurement accuracy of CGMP and SRCGMP. The video's refresh cycle (1/60 s) is set to consistent with the normal refresh cycle of the projector, and the exposure time of CCD is set as three times the refresh cycle of the video (50 ms). The measuring results are shown in Fig. 4.

The measured heart model is shown in Fig. 4a. Figure 4b is the deformed pattern of CGMP. The cutaway view in the middle row of Fig. 4b is shown in Fig. 4c. The grayscale of the captured deformed pattern ranges from 36 to 228. The reconstructed heart model with CGMP is shown in Fig. 4d. The captured deformed pattern in the SRCGMP-766 method is shown in Fig. 4e, and Fig. 4f shows the cutaway view in the middle row of it. The grayscale of the deformed pattern in the SRCGMP-766 method ranges from 31 to 765, which achieves the super-grayscale pattern capturing. It can be seen from Fig. 4c and Fig. 4f that the grayscale variation ratio in the SRCGMP-766 method is more distinct and realistic than that of the CGMP method. The heart model can be reconstructed efficiently at this condition, as shown in Fig. 4g. Further analysis of the measurement accuracy, the cutaway view in row 589 with CGMP, SRCGMP-766, and 8-PMP is shown in Fig. 4h. The experimental result with 8-step PMP (8-PMP) has high accuracy^28^ and can be regarded as quasi actual value. The profile with the SRCGMP-766 method is closer to that of the 8-PMP method. The results show that the SRCGMP-766 method has higher accuracy than the CGMP method due to realistic super-grayscale deformed patterns, which help improve the measurement accuracy originally.

### The feasibility of optimized SRCGMP

**Figure 5:**
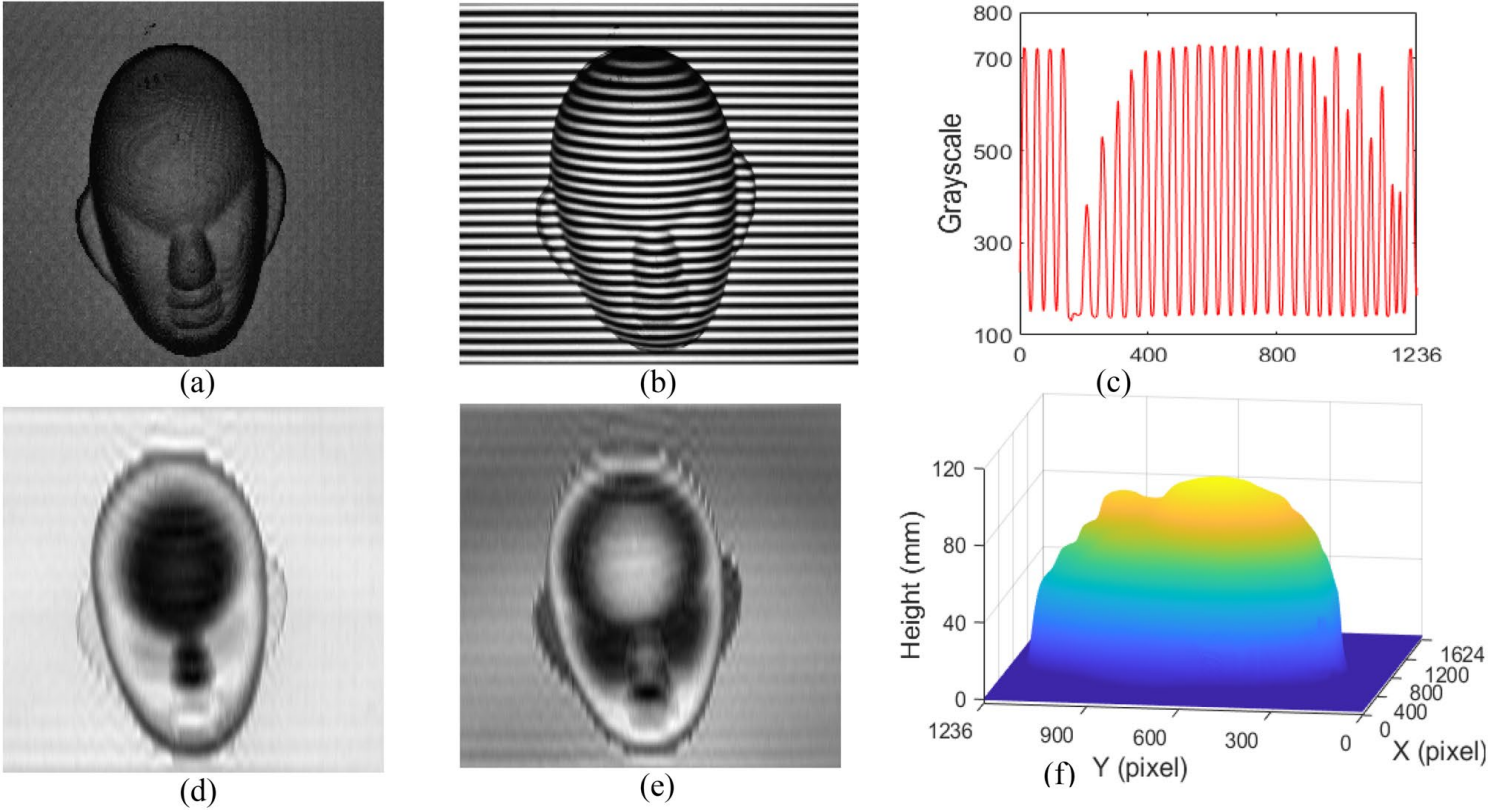
Measuring results of face model: (**a**) the measured face model; (**b**) the deformed pattern; (**c**) the grayscale at the middle row of the deformed pattern; (**d**) the 0-degree Moiré pattern; (**e**) the 90-degree Moiré pattern; (**f**) the reconstructed result.

One face model is measured to verify the feasibility of the optimized SRCGMP with 610-grayscale (SRCGMP-610) in which the grayscale is in the linear interval of the measurement system. The measuring results are shown in Fig. 5.

The measured face model is shown in Fig. 5a and the captured deformed pattern in Fig. 5b. The grayscale of the deformed super-grayscale pattern ranges from 128 to 738, which is in the linear interval of the projected intensity grayscale, and the grayscale in the middle row is shown in Fig. 5c. After extracting the AC component of the deformed pattern and multiplying it with two prepared AC components with a π/2 phase difference, respectively, the 0-degree Moiré fringe pattern and the 90-degree Moiré fringe pattern can be obtained, as shown in Fig. 5d,e respectively. The reconstructed result shown in Fig. 5f is obtained by unwrapping and phase-height mapping algorithm. The whole and detail parts of the face model, such as the nose and mouth, could be effectively reconstructed by optimized video grating.

### The measurement accuracy between CGMP, SRCGMP, and optimized SRCGMP

**Figure 6:**
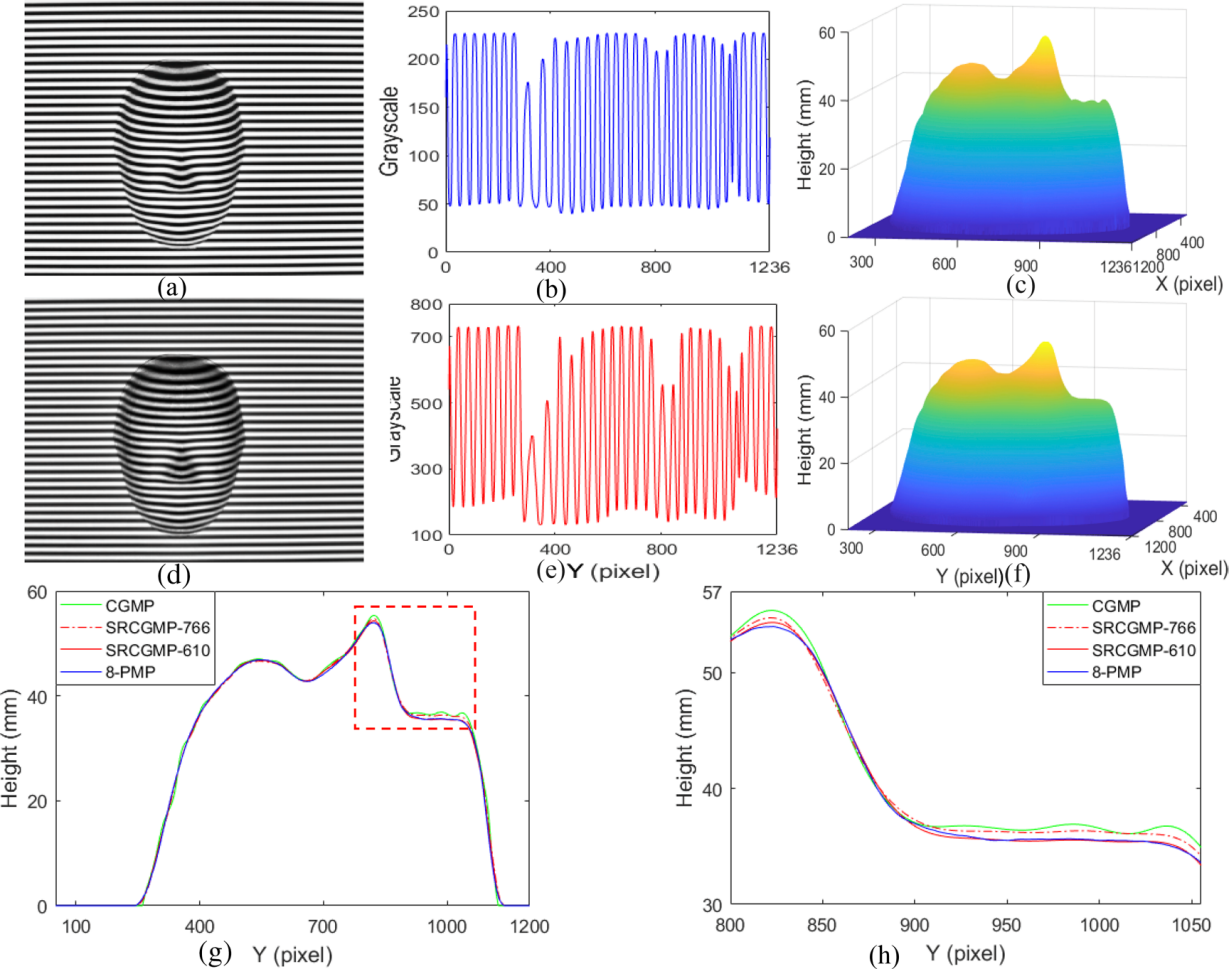
Measuring results of face mask: (**a**) the deformed pattern with CGMP; (**b**) the grayscale at the middle row of (**a**); (**c**) the reconstructed face surface with CGMP; (**d**) the deformed pattern with SRCGMP-610; (**e**) the grayscale at the middle row of (**d**); (**f**) the reconstructed face surface with SRCGMP-610; (**g**) the cutaway view at row 756 with the methods CGMP, SRCGMP-766, SRCGMP-610, and 8-PMP; (**h**) magnified view of the dashed box from (**g**).

To demonstrate the measurement accuracy of SRCGMP, one face mask is measured with CGMP, SRCGMP-766, and SRCGMP-610, compared with the 8-PMP. The measuring results are shown in Fig. 6.

Figure 6a shows the deformed pattern of CGMP. The grayscale in the middle row of the deformed pattern is shown in Fig. 6b, ranging from 36 to 228. The reconstructed face surface with CGMP is shown in Fig. 6c. Figure 6d shows the deformed super-grayscale pattern of SRCGMP-610. Figure 6e shows the grayscale of the middle row of Fig. 6d. It ranges from 128 to 738, which is in the linear interval of the measurement system. The grayscale of the SRCGMP-610 method achieves the super-grayscale deformed pattern capturing, and the grayscale variation ratio is also more distinct than the CGMP method, which is more realistic and would reduce the nonlinear effect of the projector. The reconstructed face surface with SRCGMP-610 is shown in Fig. 6f. It is evident that the reconstructed surface with the SRCGMP-610 method is smooth and shows higher fidelity. Further analysis of the measurement accuracy of the proposed method, the cutaways at row 756 of the reconstructed face surface with CGMP, SRCGMP-766, SRCGMP-610, and 8-PMP method are shown in Fig. 6g. Figure 6h is the magnified view of the dashed box from Fig. 6g. The SRCGMP-610 method has the highest measurement accuracy and is closest to the 8-PMP due to the realistic pattern and reduced nonlinear effect.

In order to further verify the measurement accuracy of the proposed method, three known heights are measured by CGMP, SRCGMEP-766, and SRCGMEP-610. The measured results are shown in Table 1. $\overline{h}$ is the mean height of the measurement results, root mean square error (MAE) denotes measurement accuracy, and root mean square error (RMS) denotes the measurement repeatability. Compared to CGMP and SRCGMP-766, SRCGMP-610 has the highest measurement accuracy.

**Table 1:** Comparison experimental results for known heights (/ mm).

*h*	6			15			23		
Method	CGMP	SRCGMP -766	SRCGMP -610	CGMP	SRCGMP -766	SRCGMP -610	CGMP	SRCGMP -766	SRCGMP -610
$\overline{h}$	6.175	6.085	6.080	15.170	15.073	15.067	23.084	23.026	23.025
MAE	0.147	0.085	0.081	0.127	0.073	0.068	0.086	0.030	0.029
RMS	0.042	0.037	0.036	0.027	0.026	0.024	0.027	0.025	0.024

### Real-time measurement

In order to verify the validity of the SRCGMP-610 method in real-time measurement, a mobile palm with gloves is used.

**Figure 7:**
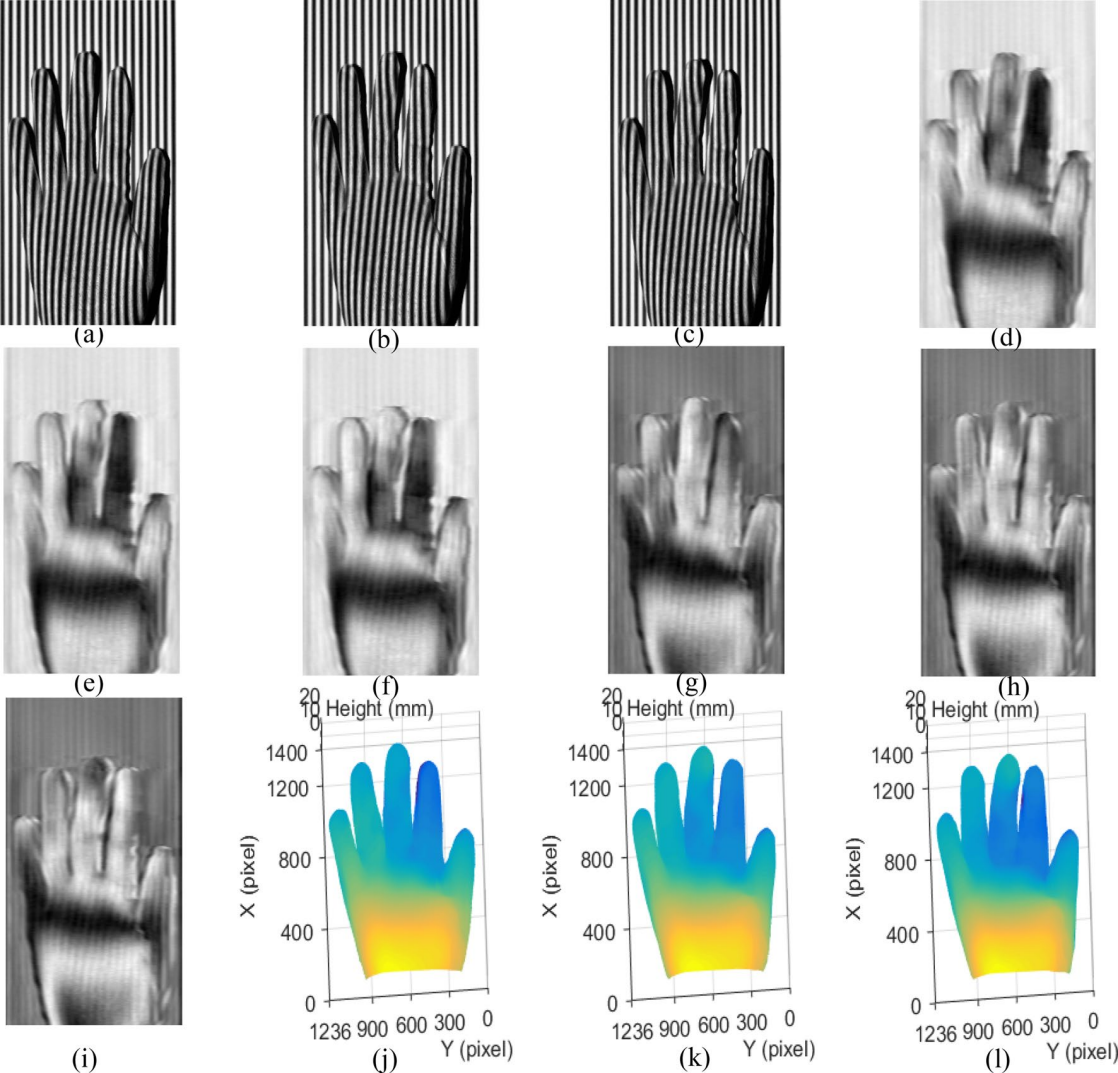
Measuring results of moving gloved palm in different states: (**a**–**c**) the deformed pattern in state one, state two and state three; (**d**–**f**) the 0-degree Moiré fringe patterns of (**a**–**c**); (**g**–**i**) the 90-degree Moiré fringe patterns of (**a**–**c**); (**j**–**l**) the reconstructed surface at three states.

The camera continuously records the deformed super-grayscale patterns and saves per pattern out of every 50 ms, which is equal to the exposure time of the camera. Three different states of the palm are used to analyze measurement results in real-time. The measuring results of the moving palm in three different states are shown in Fig. 7. Three deformed patterns with three states of the palm are shown from figure Fig. 7a–c, and the corresponding 0-degree Moiré fringe patterns and 90-degree Moiré fringe patterns are shown from Fig. 7d–i respectively. The reconstructed results of the three states palm are shown in Fig. 7j–l. It is evident that the measured palm surface at different states can be reconstructed well, which shows the validity of the real-time measurement. The real-time recorded deformed patterns of moving gloved palm (Video 1) and reconstructed results (Video 2) are included in the Supplementary Materials.

## Discussion

The super-grayscale and real-time computer-generated Moiré profilometry using video grating projection proposed in this paper have the following advantages:

### Video grating projection

The video grating synthesized by three purposefully designed 8-bit patterns is used for projecting instead of the traditional static grating in this method. The morphology of the grating has changed qualitatively. It skillfully uses the time-division multiplexing characteristics of the digital projector and the integration characteristics of the CCD camera to obtain the super-grayscale pattern.

### Super-grayscale

In this method, by projecting the designed video grating and appropriately controlling the exposure time and the gain of the camera, the super-grayscale pattern can be captured by using the time-division multiplexing characteristics of the projector and the integral exposure characteristics of the CCD camera. The super-grayscale method has a stronger linear relationship between the input gray value and output gray value and can expand the linear range by 20%, which reduces the digitized errors in reconstructed results.

### Accurate

The experimental results using the optimized video achieve both the deformed super-grayscale pattern and the whole captured intensity grayscale linear proportion to the projected intensity grayscale. That can obtain the realistic deformed pattern and reduced nonlinear effect, to originally improve the measurement accuracy.

### Real-time

This method only needs to project a repeated video onto the measured object and only needs to capture one corresponding deformed pattern for 3D reconstruction while measuring, so the single-shot feature for real-time measurement can be kept.

## Conclusion

A super-grayscale and real-time computer-generated Moiré profilometry using video grating projection is proposed by skillfully using the time-division multiplexing characteristics of the projector and the integral exposure characteristics of the CCD camera. The video grating synthesized by three purposefully designed 8-bit patterns is used for projecting instead of static grating. After projecting the repeated video onto the measured object and appropriately controlling the exposure time and the gain of the camera, the deformed super-grayscale pattern with 766-grayscale rather than the traditional 256-grayscale can be captured for reconstruction. The super-grayscale pattern is realistic and can effectively reduce digital error in computer-generated Moiré profilometry, and the proportion of the linear range of the super-grayscale pattern is expanded by 20%. The proposed method can originally improve the measurement accuracy and keep the single-shot feature for real-time measurement.

## Supplementary Information

Supplementary Video 1.

Supplementary Video 2.

## References

[1] Wu Z, Guo W, Zhang Q (2019). High-speed three-dimensional shape measurement based on shifting Gray-code light. Opt. Express.

[2] Ali I, Suominen OJ, Morales ER, Gotchev A (2020). Multi-view camera pose estimation for robotic arm manipulation. IEEE Access.

[3] Wan YY (2018). Single-shot real-time three dimensional measurement based on hue-height mapping. Opt. Commun..

[4] Melo AG, Pinto MF, Honorio LM, Dias FM, Masson JEN (2020). 3D correspondence and point projection method for structures deformation analysis. IEEE Access.

[5] Liu S (2020). A 3D spherical panorama modeling method based on double projective geometry. IEEE Access.

[6] Marrugo AG, Gao F, Zhang S (2020). State-of-the-art active optical techniques for three-dimensional surface metrology: A review Invited. J. Opt. Soc. Am. A Opt. Image Sci. Vis..

[7] Upputuri PK, Pramanik M, Nandigana KM, Kothiyal MP (2016). Multi-colour microscopic interferometry for optical metrology and imaging applications. Opt. Lasers Eng..

[8] Chen F, Brown GM, Song MM (2000). Overview of three-dimensional shape measurement using optical methods. Opt. Eng..

[9] Zhang S (2018). Absolute phase retrieval methods for digital fringe projection profilometry: A review. Opt. Lasers Eng..

[10] You Y, Shen Y, Zhang GC, Xing XW (2017). Real-time and high-resolution 3D face measurement via a smart active optical sensor. Sensors.

[11] Xiang S (2017). Hybrid profilometry using a single monochromatic multi-frequency pattern. Opt. Express.

[12] Van der Jeught S, Dirckx JJJ (2016). Real-time structured light profilometry: A review. Opt. Lasers Eng..

[13] Zhang S (2018). High-speed 3D shape measurement with structured light methods: A review. Opt. Lasers Eng..

[14] Zhang M (2017). Robust and efficient multi-frequency temporal phase unwrapping: Optimal fringe frequency and pattern sequence selection. Opt. Express.

[15] Jiang C (2015). The application of multi-frequency fringe projection profilometry on the measurement of biological tissues. Bio-Med. Mater. Eng..

[16] Wang ZZ (2014). Robust measurement of the diffuse surface by phase shift profilometry. J. Opt..

[17] Wan YY, Cao YP, Liu XR, Tao TY, Kofman J (2020). High-frequency color-encoded fringe-projection profilometry based on geometry constraint for large depth range. Opt. Express.

[18] Cao S, Cao Y, Zhang Q (2016). Fourier transform profilometry of a single-field fringe for dynamic objects using an interlaced scanning camera. Opt. Commun..

[19] Takeda M (2012). Fourier fringe analysis and its application to metrology of extreme physical phenomena: A review. Appl. Opt..

[20] Zappa E, Busca G (2012). Static and dynamic features of Fourier transform profilometry: A review. Opt. Lasers Eng..

[21] Shang W, Liu J, Ji X (2018). Study on influential factors of measurement precision using Fourier transform profilometry. Optik (Stuttgart).

[22] Liu XR, Kofman J (2019). Real-time 3D surface-shape measurement using background-modulated modified Fourier transform profilometry with geometry-constraint. Opt. Lasers Eng..

[23] Li C (2017). Computer-generated Moiré profilometry. Opt. Express.

[24] Li C (2019). High precision computer-generated moiré profilometry. Sci. Rep..

[25] Wang L (2021). Improved computer-generated moiré profilometry with flat image calibration. Appl. Opt..

[26] Wang L (2020). Orthogonal modulated computer-generated moire profilometry. Opt. Commun..

[27] Zhang H (2021). Color-encoded single-shot computer-generated Moiré profilometry. Sci. Rep..

[28] Zuo C (2018). Phase shifting algorithms for fringe projection profilometry: A review. Opt. Lasers Eng..

[29] Ma Q (2018). Intrinsic feature revelation of phase-to-height mapping in phase measuring profilometry. Opt. Laser Technol..

[30] Liu K, Wang YC, Lau DL, Hao Q, Hassebrook LG (2010). Gamma model and its analysis for phase measuring profilometry. J. Opt. Soc. Am. A Opt. Image Sci. Vis..

